# Neonatal Fc Receptor–Targeted Therapies in Neurology

**DOI:** 10.1007/s13311-021-01175-7

**Published:** 2022-01-07

**Authors:** Christopher Nelke, Marianna Spatola, Christina B. Schroeter, Heinz Wiendl, Jan D. Lünemann

**Affiliations:** 1 Department of Neurology, Medical Faculty, Heinrich Heine University Düsseldorf, Dusseldorf, Germany; 2 MIT and Harvard Medical School, Ragon Institute of MGH, Cambridge, MA USA; 3 Department of Neurology With Institute of Translational Neurology, University Hospital Münster, Munster, Germany

**Keywords:** Antibody, Therapy, Neurology, IgG, Fc

## Abstract

Autoantibodies are increasingly recognized for their pathogenic potential in a growing number of neurological diseases. While myasthenia gravis represents the prototypic antibody (Ab)-mediated neurological disease, many more disorders characterized by Abs targeting neuronal or glial antigens have been identified over the past two decades. Depletion of humoral immune components including immunoglobulin G (IgG) through plasma exchange or immunoadsorption is a successful therapeutic strategy in most of these disease conditions. The neonatal Fc receptor (FcRn), primarily expressed by endothelial and myeloid cells, facilitates IgG recycling and extends the half-life of IgG molecules. FcRn blockade prevents binding of endogenous IgG to FcRn, which forces these antibodies into lysosomal degradation, leading to IgG depletion. Enhancing the degradation of endogenous IgG by FcRn-targeted therapies proved to be a powerful therapeutic approach in patients with generalized MG and is currently being tested in clinical trials for several other neurological diseases including autoimmune encephalopathies, neuromyelitis optica spectrum disorders, and inflammatory neuropathies. This review illustrates mechanisms of FcRn-targeted therapies and appraises their potential to treat neurological diseases.

**Supplementary Information:**

The online version contains supplementary material available at 10.1007/s13311-021-01175-7.

## Introduction

A growing spectrum of neurological disorders is characterized by disease-associated immunoglobulin G (IgG) autoantibodies targeting structures of the central or peripheral nervous system as well as the neuro-muscular junction (Table 1). These disorders share several features: (1) they manifest with acute or subacute severe neurological symptoms, ranging from memory disturbances, psychosis, seizures (e.g. in autoimmune encephalitis), or demyelinating syndromes (e.g. MOG-antibody (Ab) associated disease) to muscular weakness (e.g. in AchR-associated or MuSK-associated myasthenia gravis); (2) they can co-occur with a tumour (thus, the associated syndrome is considered paraneoplastic) that is found either before or at distance of weeks to months or even years from the neurological onset; (3) the associated neuronal or glial Abs are pathogenic, as it has been demonstrated in in vitro and in animal models of passive and active immunization; and (4) immune therapies aimed at lowering Ab levels and neuroinflammation result in significant improvement of the neurological symptoms.

**Table 1:** Neurological diseases associated with Ab reactivities to autoantigen

**Antigen**	**Protein function**	**Clinical phenotype**	**Passive transfer disease model**	**Common tumor associations**
AChR (muscle)	Neurotransmitter receptor	Myasthenia gravis	+	Thymoma
AChR (ganglionic)	Neurotransmitter receptor	Autonomic dysfunction	+	Breast, prostate, lung, gastrointestinal
AMPAR	Neurotransmitter receptor	Limbic encephalitis, seizures, memory loss	+	Breast, lung, thymoma
AQP4	Water channel	NMOSD	+	Breast, lung, thymic, carcinoid, B cell lymphoma
CASPR2	Neural-glial interactions and clustering of potassium channels	Limbic encephalitis (seizures, cognitive impairment), neuromyotonia and Morvan’s syndrome, neuropathic pain	+	Thymoma
DPPX	Regulatory Subunit of Kv4.2, voltage-gated potassium channel VGKC	Confusion, hallucinations, prodromal diarrhoea, memory loss, hyperexcitability		B-cell lymphoma
D2R	Dopamin 2 receptor	Parkinsonism, chorea, psychosis, dystonia		
GABA_A_R	Ligand-gated chloride channel	Seizures, status epilepticus, psychosis	+	Thymoma
GABA_B_R	Metabotropic neurotransmitter receptor	Limbic encephalitis, seizures, memory loss	+	Lung, neuroendocrine
Glyα1R	Ligand-gated chloride channel	Encephalomyelitis, rigidity, myoclonus, seizures, stiff person syndrome	+	Ovarian, Hodgkin’s lymphoma, thymoma
IgLON5	Neuronal adhesion protein	Parasomnia, sleep apnoea, cognitive impairment, gait abnormalities		Non-Hodgkin’s lymphoma, prostate, breast
LGI-1	Involved in glutamatergic synapse development	Limbic encephalitis (seizures, cognitive impairment), faciobrachial dystonic seizures, neuromyotonia	+	SCLC, thymoma
mGluR1	Neurotransmitter receptor, G protein-coupled receptor	Cerebellar ataxia	+	Hodgkin’s lymphoma
mGluR5	Neurotransmitter receptor, G protein-coupled receptor	Confusion, psychosis, memory loss, limbic encephalitis	+	Hodgkin’s lymphoma
MOG	Member of the immunoglobulin superfamily, expressed on myelin surface	Optic neuritis, myelitis, ADEM	+	Rare
NMDAR	Heteromeric ligand-gated calcium ion channel	Encephalitis, psychosis, amnesia, behavioural abnormalities, seizures, dysautonomia	+	Ovarian teratoma, rare carcinoma, medulloblastoma in children
Neurexin 3α	Involved in synapse formation and neural adhesion	Clinical overlap with NMDAR encephalitis		
PCA-Tr/DNER	Voltage-dependant ion channel	LEMS, cerebellar degeneration, seizures, encephalopathy		SCLC
VGCC (P/Q or N type)	Voltage-gated calcium channel	Dementia, complex pain syndromes		SCLC, thymoma

*AChR* acetylcholine receptor, *AMPAR* alpha-amino-3-hydroxy-5-methyl-4-isoxazolepropionate receptor, *AQP4* aqua-porin 4, *CASPR2* contactin-associated protein-like 2, *LGI-1* leucine-rich glioma inactivated 1, *MOG* myelin oligodendrocyte glycoprotein, *NMDAR* NMDA receptor, *NMSOD* neuromyelitis optica spectrum disorders, *SCLC* small cell lung carcinoma

The neonatal Fc-receptor (FcRn) is a major histocompatibility class I–related receptor responsible for the transfer of humoral immunity from the mother to the newborn [1]. Throughout life, FcRn contributes to effective humoral immunity by recycling IgG and extending its half-life in the circulation. FcRn function can be inhibited using IgG-based and non-IgG-based agonists, by exploiting the pH-dependent binding affinity of FcRn for the IgG Fc region. Blocking FcRn function induces significant and sustained decreases in endogenous IgG levels in healthy volunteers while being safe and well-tolerated. Therapeutic FcRn blockade showed beneficial clinical efficacy in patients with generalized myasthenia gravis [2, 3] (gMG) and is a promising strategy for the treatment of Ab-mediated diseases of both the central and peripheral nervous system.

## The Neonatal Fc Receptor: from Biology to Function

FcRn was first cloned in 1994 [4], since then the structure of FcRn has been dissected in detail: FcRn is constituted by a 40-kDa α-heavy chain consisting of three extracellular domains — the α1, α2, and α3 domains — and a cytoplasmic tail connected by a transmembrane domain [4, 5]. Structurally related to MHC-I, a 12-kDa β2m-lightchain is non-covalently attached to the α-heavy chain [6, 7]. Despite FcRn sharing the MHC class I fold, the peptide-binding groove is occluded and FcRn is not thought to contribute to peptide presentation to T cells [1, 6].

Functionally, β2m knockout mice with impaired FcRn had reduced IgG levels after birth [8]. Further analysis of mice with defective FcRn revealed lower levels of IgG even as adults, providing first evidence for the role of FcRn in maintaining IgG homeostasis [1]. FcRn mRNA is not only detectable in the neonatal brush border but also in adult tissues such as liver, lung, or spleen in mice [9]. Intriguingly, decreased levels of serum IgG in FcRn defective mice were attributable to a reduced half-live suggesting a protective role for FcRn in IgG catabolism [9]. Further exploration of the consequences of defective FcRn determined that the lifespan of serum albumin is drastically reduced in FcRn deficient mice owing to albumin binding FcRn in a pH-dependent manner [10]. This effect is also observed in humans as mutations of the β2-microglobulin result in impaired FcRn function and pronounced deficiencies of serum IgG and albumin [11, 12]. The effect on albumin is less pronounced compared to IgG with FcRn deficiency leading to albumin levels of 40% in wildtype mice and of IgG of 20 to 30%, respectively [10]. Nonetheless, IgG and albumin constitute up to 90% of serum protein, thus, underpinning the pivotal role of FcRn for maintaining protein and osmotic homeostasis [1].

IgG and albumin bind to FcRn at distinct sites under acidic but not neutral pH conditions [10, 13]. FcRn-IgG interaction occurs at CH2 and CH3 and involves two central histidines, H310 and H435 [13–15]. These histidine residues are pronated at a pH of ~6 allowing for interaction of FcRn with Glu115 and Asp130. As the pH increases, pronation is lost, thus, providing an explanation for the observed dependence on an acidic Ph [13–15]. Paving the way for a comprehensive understanding of underlying pharmacokinetics, field flow fractionation of FcRn stoichiometry determined a 1:2:1 molar ratio for IgG:FcRn:albumin binding [16]. Important for translational perspectives, interaction of FcRn and its ligands differs between species. As such, murine FcRn is promiscuous given the ability of murine FcRn to bind to multiple species, including human IgG [17]. In contrast, human FcRn is limited to a range of IgG, including human, rabbit, and guinea pig IgG but not murine [17].

Expression of FcRn was recorded for numerous cells and tissues. In human tissues, expression was detected including but not limited to the placenta [18], spleen [18, 19], lungs [20, 21], intestine [18, 21, 22], liver [23, 24], kidney [24], and, most notably, vascular endothelium [25, 26]. Here, cells internalize IgG into an acidic endosomal compartment. Of note, FcRn expression differs between murine and human tissues and even among different strains of mice [27]. Translational studies should therefore be mindful when transferring results from a murine model to a human system. FcRn diverts its ligands from lysosomal degradation and recycles these molecules to the cell surface where IgG is released at a neutral pH [28, 29]. As consequence, the serum half-life of IgG and albumin are extended, explaining the surprising longevity of these proteins, ranging between 3 and 4 weeks. Blockade of FcRn recycling reduces serum IgG levels in both humans [30] and mice [31]. The contribution of FcRn-mediated IgG recycling is estimated to be 40% higher than the rate of IgG production, thus, indicating IgG recycling, and not its production, to be at the centre of IgG homeostasis.

Intriguingly, the biological consequence of FcRn activity is still evolving with recent studies in mice demonstrating that FcRn expression not only protects monomeric IgG from degradation, but circulating immune complexes (CIC) as well [30]. In humans, FcRn inhibition lead to decreased CIC levels between ~ 20 and 50% from baseline depending on the applied dosage [30]. Antigen-presenting cells, such as dendritic cells (DC), more efficiently engage T cells with antigens incorporated into IC than antigens alone [32]. FcRn is necessary for cross-presentation of IC containing IgG and, consequently, for effective engagement of T cells by antigen-presenting cells (APC) [33, 34]. This novel function is mediated by FcRn protecting antigens from lysosomal degradation. As consequence, it was shown that in mice, FcRn deficiency dampens CD8^+^ T cell stimulation by APC, providing a novel mechanism by which FcRn controls immune processes [33, 34]. A recent report delineated a further consequence of FcRn biology [35]: Intravenous immunoglobulin (IVIg) application results in supraphysiological IgG level [36] and saturates the FcRn [35]. Consequently, recycling of endogenous IgG is diminished and efficacy of exogenous IgG is amplified due to being salvaged by FcRn [35, 37]. As clinical consequence, polymorphisms in the *FCGRT* gene coding the FcRn were associated with lower levels of endogenous IgG and poor response to IVIg treatment in MG [35, 38]. In this study, patients heterozygote for the variable number of tandem repeat 2/3 (VNTR) genotype had lower IVIg efficacy than those homozygote for VNTR 3/3 [38]. However, it remains unclear whether VNTR polymorphisms were less efficacious at maintaining exogenous IgG infused by IVIg. IVIg treatment is ineffective in up to 30% of patients with neuroinflammatory diseases, such as CIDP, MG, or multifocal motor neuropathy, and FcRn biology might contribute to these outcomes. Clinical significance is discussed in further detail in [35]. FcRn function is critical for maintaining IgG and albumin homeostasis and can be harnessed to reduce levels of pathogenic IgG and to ameliorate Ab-mediated autoimmunity.

## Autoantibodies in Neurological Diseases

IgG-mediated neurological disorders represent the prime potential indications for FcRn manipulation strategies, aiming at lowering pathogenic IgG levels from the circulation through decreased FcRn-mediated IgG rescue.

### Neurological disorders mediated by autoantibodies targeting neuronal proteins

Autoimmune encephalitis associated with Abs targeting neuronal proteins represent a group of recently identified disorders that manifest with a variety of neurological symptoms, such as seizures, psychosis, memory loss, behavioural changes, altered level of consciousness to coma, dysautonomia, and movement disorders [39]. The clinical phenotype depends on age, sex, and associated tumour and on the specific Ab (Table 1). The two most common Abs found in patients with autoimmune encephalitis are those targeting the N-methyl-D-aspartate receptor (NMDAR) and the leucin-rich glioma inhibitor (LGI-1) synaptic protein. Patients with NMDAR encephalitis are usually young women (<45 years) presenting with rapidly progressive neuropsychiatric symptoms, oral dyskinesia, dysautonomia, and altered level of consciousness to coma [40]. An ovarian teratoma is often found. Removal of the tumour and immunotherapies (including PLEX, Immunoglobulins, rituximab, and cyclophosphamide) result in progressive neurologic improvement and complete recovery in the majority of patients [41]. Patients with LGI1-mediated disorder, instead, are typically older men (>65 years) with limbic encephalitis, characterized by progressive memory loss, confusion, seizures of various semiology, including generalized seizures, and the pathognomonic facio-brachial dystonic seizures, i.e. brief contractions of the ipsilateral face and arm that can occur up to 100 times/day, with preserved consciousness. A tumour is found in less than 20% of the patients, and is generally a thymoma or small-cell lung cancer [42, 43].

Overall, these autoimmune encephalitides are mainly monophasic diseases, although relapses can occur and usually manifest with similar symptoms to the initial presentation.

The pathogenicity of these Abs, that are mainly IgG1 (e.g. NMDAR Abs) or IgG4 (e.g. LGI1, CASPR2 Abs), has been demonstrated in neuronal cultures and, for some of them, also in mouse models. Treatment of primary cultures of neurons with patients-derived Abs results in neuronal dysfunction through different mechanisms, including cross-linking and internalization (e.g. NMDAR Abs [44, 45]), disruption of protein–protein interaction (e.g. LGI1 [46]), or blocking of receptor function (e.g. GABA_B_R). Also, NMDAR Abs from affected patients, when infused intraventricularly into mice brains, cause altered memory and behaviours, NMDAR dysfunction and altered long-term synaptic plasticity [47, 48]. Similarly, intraventricular infusion of patients-derived LGI1 Abs in mice prevented binding of LGI1 to its cognate proteins ADAM23 and ADAM22, caused neuronal hyperexcitability, decreased synaptic plasticity, and memory deficits [49]. Importantly, in both models, these molecular and behavioural effects were reversible upon removal of the Abs, reflecting the valuable response to Ab-depleting therapies observed in humans, and supporting a direct pathogenic role of these Abs in a murine model.

### Neurological Disorders Mediated by Autoantibodies Targeting Glial or Myelin Proteins

In the last decades, Abs targeting the MOG protein and the water channel AQP4 have been identified in patients with inflammatory demyelinating disorders, defining clinically and immunologically distinct diseases from multiple sclerosis. AQP4 Abs have been associated with neuro-myelitis optica spectrum disorder (NMOSD), which manifests as severe attacks of optic neuritis, longitudinally extensive myelitis, diencephalon, and brainstem involvement, including area postrema syndrome (nausea, vomiting, incoercible hiccup). The clinical phenotype of MOG-associated disorder is age-dependent: young children mostly present with acute disseminated encephalomyelitis (ADEM), whereas older children and adults manifest with optic neuritis or, less frequently, with extensive myelitis or encephalitis [50]. Although MOG-associated disorder can have a monophasic course (which is rarely the case for AQP4-associated NMOSD), 70% of the patients experience relapses [51]. Persistent detection of high MOG titers has been associated with a higher risk of relapse in children with ADEM [51, 52].

In AQP4 Ab-associated optic neuritis, optic nerve damage depends on the severity of each attack; conversely, the nerve injury associated with MOG Abs seems to be related mainly to the frequency of attacks. Overall, outcome is generally better in MOG-associated than in AQP4-associated optic neuritis [53]. Timely treatment with steroids, intravenous immunoglobulins and immunosuppressive therapy (such as azathioprine, mofetil mycophenolate, or rituximab) is essential to improve recovery from the onset attack and to lower the risk of relapses in both diseases [54, 55].

Unlike neuronal Abs that can be found exclusively in the CSF of patients with seronegative autoimmune encephalitis, both MOG and AQP4 Abs are mostly found in serum with Abs mainly constituted by the IgG1 subclass. Their pathogenic effects have been extensively demonstrated in both in vitro studies, mainly through IgG1-mediated complement activation and natural killer cytotoxicity [56, 57], and in animal models. Also, AQP4-IgG can activate astrocytes and induce inflammatory changes even in the absence of complement [58]. In experimental autoimmune encephalomyelitis (EAE) models with mice immunized with MOG, the clinical phenotype depends on the balance between T cells and MOG-Ab titers, where a high cellular component drives an ADEM phenotype, whereas an excess of MOG Abs leads to an optico-spinal disease [59]. However, unlike AQP4 Abs that cause NMOSD pathology in mice infused with patients’ Abs [60], intracerebral injection of human MOG Abs do not cause disease in rodents, suggesting an indirect role for MOG Abs, as supported by observations that MOG-reactive T cells induce higher levels of inflammation in presence of MOG Abs [61]. However, one must be prudent when interpreting these results as differences between species are likely to affect results. Ample clinical evidence and the efficacy of Ab-depleting therapies [62, 63] underline the pathogenic potential of Abs directed against structures of the CNS. Nonetheless, despite convincing evidence in animal models, for large parts of the neurological Ab-spectrum concluding evidence of pathogenicity is lacking in humans.

### Neurological Disorders Mediated by Autoantibodies Targeting the Neuro-muscular Junction

Abs targeting AChR, MuSK, or other functionally related molecules (such as the lipoprotein receptor–related protein 4 [LRP4]) induce myasthenia gravis, i.e. a chronic autoimmune disease characterized by weakness of skeletal muscles, which can be generalized or involve only few muscular units, often including the extraocular muscles, with diplopia and ptosis. The weakness typically increases with muscle use (fatigability) and fluctuates during the day and from day to day. Although the disease can initially be localized, for instance in the purely ocular forms, within the first 2 years, it becomes generalized in more than 80% of the patients [64]. Overall, myasthenia gravis is associated with a thymoma or a thymic hyperplasia in less than a quarter of the patients [65, 66], although its prevalence increases in older individuals. The type of Ab determines the age of onset (for example patients with AChR Abs typically have a bimodal early-onset or late-onset disease), frequency of associated thymoma (very common in the forms associated with AChR Abs, exceptional in those associated with MuSK Abs), clinical phenotype and severity (anti-MuSK myasthenia gravis has often more severe weakness and involves bulbar muscles more often than anti-AChR forms), and response to treatment (MuSK-associated forms usually have less favourable response to symptomatic treatment and immunotherapies, see below). Abs targeting AChR are of IgG1 and IgG3 subclass, and have been demonstrated to cause muscular weakness through distinct mechanisms [67]: cross-linking and internalization of their target, resulting in depletion of AChR from the synaptic cleft [68]; complement-mediated destruction of the post-synaptic muscular membrane [69]; and competition with acetylcholine on the ACh binding site of the AChR, preventing activation of the receptor [70]. In rat models obtained by active immunization with AChR induce experimental autoimmune myasthenia graviss (EAMG) [71]. By contrast, MuSK and LRP4 Abs are of IgG4 class, which are unable to fix complement and only weakly bind Fc receptors on the immune cells. In these cases, pathogenic mechanisms are thought to be caused by Abs interfering with interaction between their targets and binding partners (e.g. between MuSK and LRP4 or between agrin and LRP4) [67].

The main therapeutic strategies for myasthenia gravis include (1) symptomatic treatment, which aims to potentiate the neuromuscular transmission through the use of acetylcholinesterase inhibitors, and (2) immunosuppressive therapy (including prednisone/prednisolone and azathioprine or mycophenolate mofetil, or rituximab) to induce a durable remission of symptoms over time. Given that the thymus is considered to play a key role in inducing AChR Ab production, total thymectomy is recommended not only in patients with myasthenia gravis and thymoma, but it has been shown to be beneficial even in those without thymoma [72, 73].

## Harnessing FcRn Biology to Treat Neurological Diseases

**Fig. 1:**
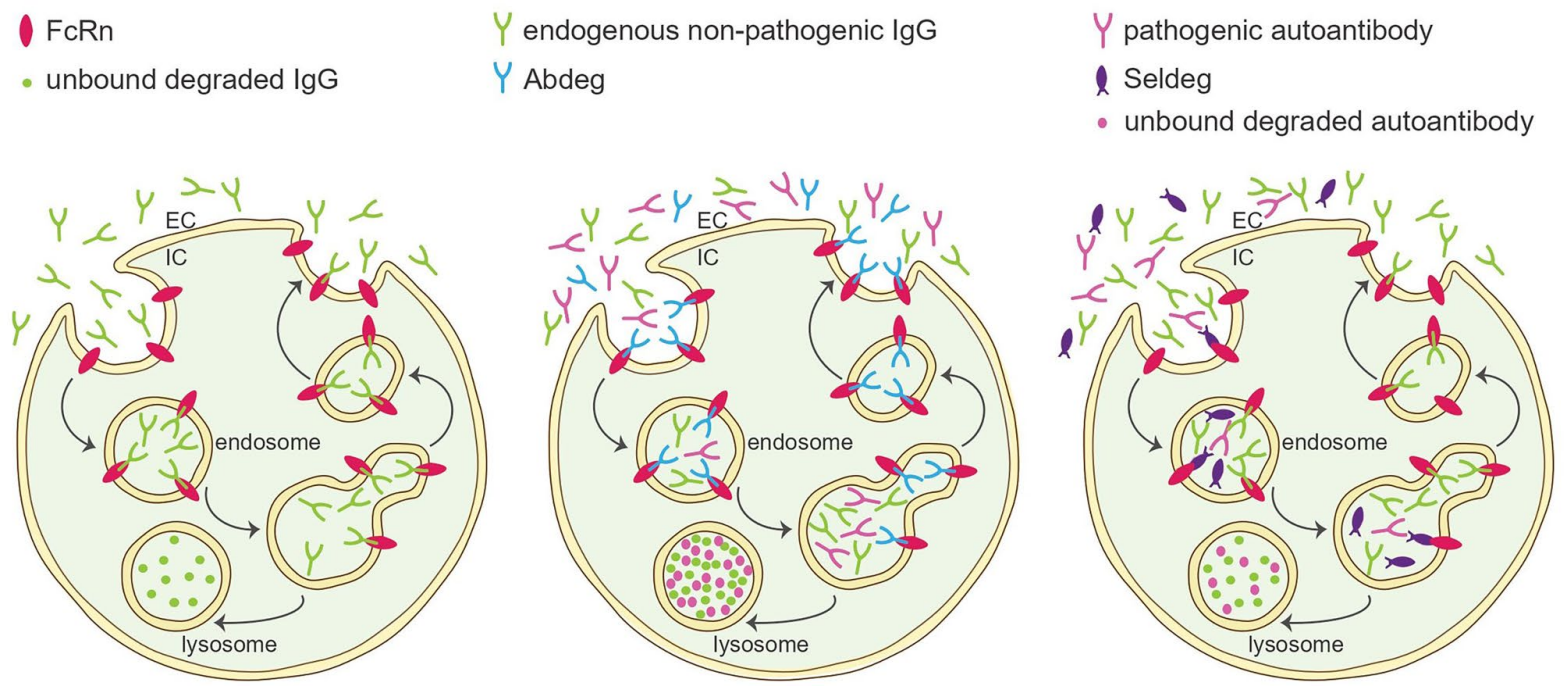
Harnessing FcRn function to deplete pathogenic IgG antibodies. Left: Physiological FcRn function. Endogenous IgG molecules bind FcRn and are prevented from entering lysosomal degradation, thus, extending their half-life. Middle: Function of Abdegs. Abdegs bind FcRn and prevent IgG-FcRn interaction. Endogenous non-pathogenic and pathogenic IgG are directed towards lysosomal degradation. Abdegs are recycled to the cell surface. Right: Function of Seldegs. Seldegs bind pathogenic autoantibodies. Seldeg-IgG complexes then bind FcRn and facilitate receptor-mediated internalization and lysosomal degradation of bound IgG

Based on the established efficacy of plasmapheresis (PLEX) or IVIg for amelioration of several Ab-mediated diseases, harnessing FcRn might allow to elegantly achieve comparable efficacy while avoiding adverse effects and reducing supply issues. Indeed, therapeutic Abs targeting FcRn, commonly referred to as Abdegs (ab that enhance IgG degradation), are effective at reducing serum IgG levels [34]. Abdegs are engineered to bind FcRn at high affinity, effectively outcompeting endogenous IgG and promoting rapid catabolism (Fig. 1).

Abdegs are under investigation for a number of autoimmune conditions, most notably MG, neuromyelitis optica spectrum disorder (NMOSD) or chronic inflammatory demyelinating polyneuropathy (CIDP).

We will first dissect the clinical landscape of FcRn targeted therapies in the context of MG as a model disease for Ab-mediated autoimmunity. MG constitutes the major autoimmune disorder affecting the neuromuscular junction. The pathophysiological hallmark of MG are pathogenic IgG Ab directed against structures of the postsynaptic membrane [74]. In recent years, abolition of complement emerged as valuable treatment strategy for MG as evidenced by the results of the phase II REGAIN trial [75] investigating the C5 inhibitor eculizumab. It is important to note that complement therapy is limited to IgG Ab known to activate complement, such as anti-acetylcholine receptor-Ab (anti-AChR-Ab) investigated in the REGAIN trial. While the majority of patients (~ 85%) are anti-AChR-Ab positive, ~ 5 to 8% of patients display anti-muscle-specific tyrosine kinase (MuSK)-Abs [76]. This distinction is important as anti-MuSK-Ab are IgG4 and, thus, unlikely to induce complement-mediated damage to the neuromuscular junction [77]. As consequence, complement targeted therapies are considered unfit for anti-MuSK-Ab MG. Given that MG patients with anti-MuSK-Ab are more often affected by a disease course refractory to standard treatments [76], therapeutic options for these patients constitute an unmet clinical need. Abdegs depleting pathogenic Ab might therefore be valuable for MG patients as a whole, and anti-MuSK-ab positive patients in particular.

Indeed, Abdegs are under investigation in preclinical as well as in clinical studies for treatment of MG. An overview over current FcRn therapies is given in Table 2. In the following chapter, we will discuss FcRn therapies potentially becoming available for treatment of MG. First, Efgartigimod (ARGX-113) is modified human IgG1-derived Fc fragment constructed to bind and antagonize FcRn at neutral and acidic pH [2, 78]. Although technically not an Abdeg, Efgartigimod has been investigated in cynomolgus monkeys with a dosage of 20 mg/kg resulting in a maximum reduction of endogenous IgG levels of 75%. Following these promising results, a phase I study evaluated single ascending doses and multiple ascending doses in a first-in-human study [78]. The single ascending dose regime led to a decrease in IgG1 of 42% at a maximum dose of 50 mg/kg, while multiple doses led to a more pronounced decrease of IgG1 reaching 78% at a maximum dose of 25 mg/kg. Importantly, adverse effects were negligible with no serious events observed. These findings were translated to a phase II study reporting first evidence supporting antagonism of FcRn as potential treatment for MG [2]. This phase II study investigated Efgartigimod for generalized MG (gMG) in a randomized, double-blinded, placebo-controlled trial with a 1:1 assignment of patients. Patients received 10 mg/kg of Efgartigimod or placebo for analysis of safety as primary endpoint, while efficacy was included as secondary endpoint. A total of 24 patients (12 patients per group) were included, corroborating previous results with no serious or severe adverse effects in either group [2]. Moreover, Efgartigimod was efficacious for treatment of gMG and improved clinical readouts, including the activities of daily living (ADL)-score and quantitative MG (QMG)-score. Improvements were achieved 1 to 2 weeks after the last dose with a maximum decrease of −5.7 points and −4.4 points for the QMG- and ADL-scores, respectively. Importantly, this phase II study was restricted to anti-AChR-ab MG patients and no anti-MuSK-ab patients were included. Efgartigimod is currently under investigation in a phase III study (NCT03669588) applying a primary endpoint assessing the response of MG-ADL at 8 weeks for anti-AChR-ab positive MG patients. Besides Efgartigimod as an example for an engineered Fc fragment, anti-FcRn-abs are currently being developed. Here, Rozanolixizumab, Nipocalimab, Orilanolimab, and RVT-1401 are worth mentioning. Of these, Rozanolixizumab is engineered an IgG4 monoclonal Ab (mAb) engineered to bind FcRn at high affinity [79]. In a phase I study aimed at evaluating the safety profile, 36 patients were treated with Rozanolixizumab and 13 with placebo. Treatment was overall well tolerated with three patients reporting headaches and one patient reporting back pain at the highest study dose of 7 mg/kg [79]. Following the pronounced reduction of serum IgG in this phase I study, results from a phase II study were recently reported [80]. Here, patients were randomized 1:1 to Rozanolixizumab and placebo (days 1 to 29) and re-randomized in a second treatment period (days 29 to 44), followed by an observation period (days 44 to 99). Treatment with Rozanolixizumab did not reach statistical significance for the change to baseline QMG-score as primary endpoint. However, change to MG-ADL-score was significant and a phase III study is ongoing [80]. Interestingly, the study attempted to evaluate anti-MuSK-ab patients; however, only one patient was included, forestalling a conclusive statement regarding the efficacy of Abdegs for this serological subgroup. Succinctly, we observe that FcRn antagonism leads to a sharp reduction in serum IgG and sustained clinical efficacy, while adverse effects were rare.

**Table 2:** Treatment strategies harnessing FcRn function

**Treatment**	**Disease/Indication**	**Treatment strategy**	**Reference or ClinicalTrials.gov identifier**
Efgartigimod (ARGX-113)	MG CIDP	Antagonism of FcRn (IgG1-derived Fc fragment with high affinity for FcRn)	[2, 78]
Nipocalimab	MG	Antagonism of FcRN (Abdeg, aglycosylated IgG1 mAb against FcRn)	NCT04951622 (recruiting)
Rozanolixizumab	MG CIDP	Antagonism of FcRn (Abdeg, humanized mAb against FcRn)	[80, 96]
Orilanolimab (SYNT001)	MG	Antagonism of FcRn (Abdeg, IgG4 mAb against FcRn)	[30]
CSL730/M230	Unknown	Antagonism of FcRn (Abdeg, IgG1 Fc multimer against FcRn)	NCT03375606 (terminated) NCT04446000 (ongoing)
Batoclimab (RVT-1401)	MG CIDP NMSOD	Antagonism of FcRn (Abdeg, mAb against FcRN)	[111] NCT04346888 (completed)
MOG-Seldeg	NMSOD	Selective degradation of pathogenic Abs (Seldeg, recombinant MOG protein linked to a human IgG1-derived Fc fragment with high affinity for FcRn)	[81, 82]
Ravulizumab	MG NMSOD	Increased half-life of anti-C5-mAb (mAb with high affinity for FcRn, created by modifying Eculizumab)	[87] NCT03920293 (MG, active, not recruiting) NCT04201262 (NMSOD, active, not recruiting)
Satrulizumab	NMSOD	IgG2 mAb against IL-6, binds FcRn for recycling and prolonged half-life	[88] NCT02028884 (active, not recruiting)

*Ab* antibody, *abdeg* antibody-based FcRn inhibitor, *CIDP* chronic inflammatory demyelinating polyneuropathy, *MG* myasthenia gravis, *NMSOD* neuromyelitis optica spectrum disorders, *seldeg* selective degradation

Given the promising effects observed for MG, indications for Abdegs are likely to evolve. Besides MG, the phase II ADHERE trial (NCT04281472) investigates Efgartigimod for treatment of CIDP, including 400 participants in a randomized, placebo-controlled setting. Similarly, Rozanolixizumab (NCT03861481) and the fully human anti-FcRn mAb Batoclimab are under investigation in clinical trials for treatment of CIDP. Abdegs might provide a novel mechanism capable of ameliorating Ab-mediated disease while curtailing costs and risks associated with standard therapies. Nonetheless, long-term outcomes of IgG depletion remain unknown and will likely shape our understanding of FcRn modulation in the future.

A potential drawback of Abdeg technologies is the unselective depletion of IgG, including those mediating host defence. To overcome this caveat, research teams engineered a novel class of agents that selectively clear antigen-specific Abs by exploiting the FcRn mechanism [81]. Indicating their ability to facilitate selective degradation, these agents were termed Seldegs (Abs designed for selective degradation of pathogenic antibodies) [81, 82]. Myelin oligodendrocyte glycoprotein (MOG) participates in the myelination of nerves and is a characteristic target for autoreactive Abs in mouse and humans [83]. In human disease, anti-MOG-ab status is discussed to constitute a clinically distinct subset of patients in seronegative Neuromyelitis Optica Spectrum Disorder (NMSOD) [84]. As proof-of-concept, MOG-Seldegs were engineered to display recombinant MOG protein linked to a human IgG1-derived Fc fragment. In contrast to naturally occurring IgG, this Fc fragment binds FcRn at near neutral pH leading to rapid receptor-mediated internalization and degradation [81]. To test the therapeutic potential of Seldegs, experimental autoimmune encephalitis (EAE) mice with disease exacerbation due to transfer of MOG-specific antibodies derived from multiple sclerosis patients were treated with MOG-Seldegs [82]. Indeed, application of MOG-Seldegs resulted in selective depletion of pathogenic Abs [81] and amelioration of disease in the EAE-model [82]. While Seldegs are a novel concept, this elegant technology might hold advantages compared to Abdegs as the former selective deplete pathogenic IgG and largely maintain immune homeostasis. However, application of Seldegs requires intimate knowledge of the pathogenic IgG mediating autoimmunity, likely restricting the use of Seldegs to a limited number of indications.

The FcRn mechanism could be used therapeutically in two ways: (i) to reduce the half-life of pathological antibodies or (ii) to increase the half-life of therapeutic antibodies. Abdeg and Seldeg technologies target the first mechanism. Many therapeutic mAbs have proven efficacious for a number of neurological indications, i.e. Eculizumab inhibits complement activation in MG by inhibiting C5 [75], while Tocilizumab targets IL-6 for treatment of NMSOD [85]. However, therapeutic mAbs undergo continuous endocytosis and lysosomal degradation, effectively limiting their half-life and biological activity [86]. Increasing the dissociation rate of mAbs and their target molecule at an acidic pH was hypothesized to promote recycling of the unbound mAb into the circulation. This recycled mAbs could then bind new targets and extend its biological activity. Indeed, two examples of agents harnessing this mechanism are Ravulizumab targeting C5 [87] and Satrulizumab targeting IL-6 [88], respectively.

Approximately two-thirds of NMSOD patients have detectable IgG Abs against aquaporin-4 (AQP-4) [89]. IL-6 levels are increased in cerebrospinal fluid of NMSOD patients, particularly in disease relapses, with IL-6 promoting B cell maturation into AQP-4 secreting plasmablasts [89]. To target IL-6 as driver of disease, Satralizumab, a humanized mAb recognizing membrane-bound and soluble IL-6 receptor, was developed. Intriguingly, Satralizumab employs FcRn recycling to extend its half-life by dissociating from IL-6 in a pH-dependent manner [88]. The efficacy of Satralizumab for ameliorating NMSOD a phase III, randomized, double-blinded, placebo-controlled trial investigated Satralizumab as addon to baseline immunosuppressants [88]. Exaactly 83 patients were assigned 1:1 with 41 patients receiving 120 mg Satralizumab administered subcutaneously and 42 patients receiving placebo. Both AQP-4 positive and negative NMSOD were included with the first relapse defined as primary endpoint. Here, Satralizumab-treated patients had significantly fewer relapses with 8 patients (20%) as compared to 18 patients (43%) receiving placebo. Interestingly, pain and fatigue scores did not differ between Satralizumab and placebo [88].

For MG, Eculizumab has proven effective for treatment-refractory disease [75]. However, the terminal half-life of ~ 11 days requires dosing every two weeks to maintain treatment efficacy [90]. To improve pharmacokinetics, Ravulizumab was engineered by incorporating “histidine switches” into the complementarity-determining regions of Eculizumab [91]. Thereby, the dissociation rate of Ravulizumab and C5 is increased, cumulating in an extended duration of mAb activity [91]. The efficacy of Ravulizumab has been investigated in a phase 3 study for treatment of gMG [92]. According to the sponsor, the primary endpoint of change from baseline MG-ADL was met and maintained for a total of 52 weeks [92]. The final report is pending; however, these preliminary results underline the feasibility of FcRn modulation for improvement of mAb technologies.

## Safety and Tolerability

Removal of serum proteins by PLEX and even by immunoadsorption is unselective, while FcRn-targeted therapeutics are confined to reduction of IgG and albumin, possibly providing fewer off-target effects. As of now, safety concerns of Abdegs are focused on the consequences of IgG depletion [93]. Clinical trials of Abdegs have demonstrated substantial reductions of serum IgG up to ~ 70% from baseline [94]. Nonetheless, therapeutic application of Abdegs did not result in increased rates of infectious complications in clinical trials [2, 95, 96]. Function of other Ig, including IgA and IgM, and immune cell homeostasis and complement appear to be undisturbed in response to Abdeg treatment [95, 96], pointing to maintenance of immunological host defence during therapy. Apart from infections, frequently reported adverse effects in patients treated with Abdegs, such as efgartigimod or rozanolixizumab, were headaches, upper respiratory tract infections, and leukocytopenia or lymphocytopenia [2, 80] — all of which were not severe. Taken together, the current knowledge regarding FcRn targeted therapeutics suggests that adverse effects are mostly mild with headaches as most frequent symptom and manageable with standard treatments.

However, it is important to note that there is a lack of long-term studies investigating effects of IgG depletion. Given the mechanism of action, infectious complications arising from IgG deficiency remain the primary concern for treatment. To better understand long-term outcomes, we may consider IgG depletion as a type of secondary immunodeficiency (SID) [97]. As opposed to primary immunodeficiencies arising from genetic defects, SID may be acquired upon immunosuppressant treatment [98]. As a multifactorial entity, the continuum of immunodeficiency varies in severity, ranging from high risks for infections as observed in lymphoid malignancies [99] to more benign forms as seen for genetic defects resulting in impaired IgG production [100]. As opposed to lymphoid malignancies or B cell depleting therapies such as rituximab [101], FcRn therapies are discrete in action only depleting IgG, while other Ig-subclasses as well as immune cell subsets remain functional. Translating these considerations into a clinical setting, patients receiving FcRn treatments appear unlikely to be at risk for pathogens, that require immune cells for clearance. As such, opportunistic infections by encapsulated bacteria, including *Neisseria meningitidis* or *Streptococcus pneumoniae*, are resolved by the innate immune response [102], with Ab-mediated immunity only playing a minor role [103]. In contrast, IgG serves distinct immune functions, such as mediating mucosal protection or viral clearance [95, 104]. Therefore, IgG-depletion by FcRn therapies might impose minor, but distinct infectious risks on patients.

Besides, FcRn function is evolving across human tissues. As such, FcRn is expressed in the microvascular epithelium constituting the blood–brain-barrier (BBB) [105, 106]. These studies, albeit in mice, detected FcRn expression and suggest that FcRn mediates IgG efflux from the brain into the bloodstream via transocytosis [105, 107]. However, a number of studies contrast this data and observed no meaningful role of FcRn for IgG trafficking [108, 109]. Recently, an in vitro study of human pluripotent stem cells concluded that Ab trafficking from the brain occurs independently of FcRn [110]. Given the important role of BBB integrity in neuroinflammatory disease, it is important to note that FcRn inhibition might potentially affect cerebral IgG homeostasis and, thus, long-term outcomes. Studying FcRn in the context of BBB function, particularly in a human model, is important to dissect advantageous and disadvantageous effects of FcRn modulation. Our understanding of the adverse effects profile of FcRn-targeted therapies will likely evolve over time and long-term studies are warranted to pinpoint immunological risks associated with treatment.

## Outlook

Targeting the FcRn is a novel and promising approach for the treatment of a number of Ab-mediated neurological diseases due to selective IgG depletion and can additionally be used to extend the half-life and efficacy of therapeutic mAbs. FcRn blockade is particularly intriguing as it does not result in general immune suppression, in contrast to many conventional therapies in routine clinical use, while the long-term safety of recurrent IgG depletion cycles remains to be addressed. As IgG molecules are the preeminent effector proteins of the immune system and recruit and activate leukocytes through Fc interactions with Fc receptors (FcRs) expressed by innate immune cells and B cells, the removal of pathogenic IgG molecules by FcRn targeted therapies likely affects both humoral and cellular immunity. Along these lines, a third of patients with gMG positive for AChR Abs who responded to efgartigimod in experienced clinical improvement that lasted more than 12 weeks [2]. At this time point, IgG levels had returned to baseline, suggesting that a sustained reprogramming of the pathogenic humoral immune response or restoration of immune regulatory networks occurred, at least in a subset of participants. To improve our understanding of FcRn therapies’ mechanisms of action beyond simple IgG depletion, studies that apply high-dimensional deep immunophenotyping approaches to high-quality biological samples from carefully characterized patient cohorts to more completely understand changes in the ‘immunome’ and correlate these changes with clinical outcomes will be instrumental. Targeted combination therapies with distinct or complementary mechanisms, such as FcRn targeted therapies in combination with complement inhibitors, could determine whether they provide additional efficacy with favorable safety over existing regimens. Such insights will help to define the clinical significance and guide the optimal use of FcRn targeted treatments as a therapeutic strategy in neurology.

## Supplementary Information

Below is the link to the electronic supplementary material.

Supplementary file1 (DOCX 34 KB)

Supplementary file2 (DOCX 34 KB)

Supplementary file3 (DOCX 34 KB)

Supplementary file4 (DOCX 35 KB)
